# GPs’ experiences of children with anxiety disorders in primary care: a qualitative study

**DOI:** 10.3399/bjgp17X693473

**Published:** 2017-10-24

**Authors:** Doireann O’Brien, Kate Harvey, Bridget Young, Tessa Reardon, Cathy Creswell

**Affiliations:** School of Psychology and Clinical Language Sciences, University of Reading, Reading.; School of Psychology and Clinical Language Sciences, University of Reading, Reading.; Institute of Psychology, Health and Society, University of Liverpool, Liverpool.; School of Psychology and Clinical Language Sciences, University of Reading, Reading.; School of Psychology and Clinical Language Sciences, University of Reading, Reading.

**Keywords:** anxiety disorders, barriers, child mental disorders, general practice, health services accessibility, primary health care

## Abstract

**Background:**

Anxiety disorders have a median age of onset of 11 years and are the most common emotional disorders in childhood; however, a significant proportion of those affected do not access professional support. In the UK, GPs are often the first medical professional that families see so are in a prime position to support children with anxiety disorders; however, currently there is little research available on GPs’ perspectives on and experiences of supporting children with these disorders.

**Aim:**

To explore the experiences of GPs in relation to identification, management, and access to specialist services for children (<12 years) with anxiety disorders.

**Design and setting:**

Twenty semi-structured interviews were conducted with GPs in primary care throughout England.

**Method:**

GPs reflected a diverse group in relation to the ethnic and socioeconomic profile of registered patients, GP age, sex, professional status, previous engagement with research, and practice size and location. Purposive sampling was used to recruit GPs until theoretical saturation was reached. Data were analysed using a constant comparative method of thematic analysis.

**Results:**

Data from 20 semi-structured interviews were organised into three themes: decision making, responsibility, and emotional response, with an overarching theme of GPs feeling ill equipped. These themes were retrospectively analysed to illustrate their role at different stages in the primary care process (identification, management, and access to specialist services).

**Conclusion:**

GPs feel ill equipped to manage and support childhood anxiety disorders, demonstrating a need for medical training to include greater emphasis on children’s mental health, as well as potential for greater collaboration between primary and specialist services.

## INTRODUCTION

Anxiety disorders are common across the lifespan, with a lifetime prevalence of 28.8%.^1^ At least half of these disorders start before age 11,^1^ and are the most common emotional disorder in childhood,^2^^–^4 with worldwide prevalence rates of 6.5%.^5^ They are associated with an increased risk of subsequent mental health problems, substance misuse, and poor educational attainment,^6^^–^9 and have a high economic and societal burden.^10^^,^^11^ As such, there is a need for early access to evidence-based intervention. Effective treatments for childhood anxiety disorders exist;^12^ however, only a minority of affected children access this support.^13^^–^15

Typically, a child’s problem must be recognised by the parent/caregiver and subsequently by a medical professional.^16^ In the UK, GPs are often the first medical professional that families see, so are ideally positioned to support families themselves as well as being increasingly seen as ‘gatekeepers’ to specialist Child and Adolescent Mental Health Services (CAMHS).^17^^–^19 Two-thirds of British children see their GP at least once a year,^20^ and there has been a steady rise in the number of children and young people presenting in primary care with mental health difficulties.^21^^–^26 GPs are in a position to develop strong relationships with families, in a non-stigmatising setting.^27^^,^^28^ Many GPs report a lack of confidence in their competence and skills in child and adolescent mental health, reflecting a need for further training,^29^^,^^30^ and believe that their role in this area requires further research and definition.^31^

Childhood anxiety disorders may present a particular challenge for GPs because of their broad clinical presentation^32^ and the common reliance on parental recognition,^33^ which may reduce clinician confidence in identification and management.^34^ As such, the present study aimed to explore the experiences of GPs in England in identification, management, and accessing specialist services for childhood anxiety disorders, if appropriate.

## METHOD

### Study design

The study comprised individual semi-structured telephone interviews with 20 GPs located in different practices throughout England (Table 1). All interviews were conducted by one researcher, who received training in qualitative research methods. Data were collected from April 2015 to October 2015 and analysed using thematic analysis.

**Table 1.** Demographic characteristics of GPs and their patient populations

**ID**	**Geographical location**	**Practice size**	**Sex**	**Years qualified**	**Psychiatry rotation**	**Status**	**Area**	**Socioeconomic status of practice^a^**	**Ethnicity estimate^b^**	**Research active^c^**	**Childhood anxiety disorder presentation^c^**	**CAMHS use^c^**
1	Urban	Large	Female	13	No	Partner	North England	High	n/a	No	Frequent	Frequent
2	Urban	Small	Male	10	No	Partner	Wessex	High	n/a	n/a	Never	Never
3	Rural	Small	Female	10	Yes	Principal	North England	Medium	<1%	Yes	Infrequent	Infrequent
4	Rural	Small	Male	18	Yes	Partner	Wessex	High	<1%	Yes	Infrequent	Never
5	Rural	Small	Male	26	No	Partner	Yorkshire	High	<1 %	Yes	Infrequent	Infrequent
6	Suburban	Medium	Female	21	No	Partner	Wessex	High	2.5%	No	Infrequent	Infrequent
7	Suburban	Small	Male	7	No	Partner	Wessex	High	<1%	Yes	Infrequent	Infrequent
8	Urban	Medium	Female	12	Yes	Principal	NW London	Medium	34.7%	Yes	Frequent	Frequent
9	Suburban	Small	Female	19	No	Partner	NW London	Medium	45.2%	Yes	Infrequent	Infrequent
10	Suburban	Large	Female	11	No	Partner	Wessex	High	1.7%	No	Frequent	Frequent
11	Urban	Large	Female	23	Yes	Salaried GP	TV&SM	Medium	15.5%	No	Frequent	Frequent
12	Rural	Small	Male	6	No	Partner	TV&SM	High	4.2%	Yes	Infrequent	Infrequent
13	Urban	Medium	Female	16	Yes	Partner	NW London	Medium	n/a	n/a	Frequent	Frequent
14	Urban	Medium	Male	29	Yes	Partner	North Thames	Low	n/a; GP reported 70% of people in this area come from BME groups	No	Infrequent	Infrequent
15	Suburban	Large	Female	17	No	Partner	West England	High	0	Yes	Frequent	Frequent
16	Urban	Medium	Male	22	Yes	Partner	NW Coast	Low	n/a	n/a	Infrequent	Infrequent
17	Rural	Medium	Male	6	No	Salaried GP	NW Coast	Medium	7.9%	Yes	Infrequent	Infrequent
18	Urban	Medium	Female	9	Yes	Partner	NW Coast	Low	1.7%	No	Frequent	Infrequent
19	Urban	Medium	Female	14	Yes	Partner	NW Coast	High	0	Yes	Frequent	Frequent
20	Urban	Medium	Male	2	No	Principal	NW Coast	Medium	5%	Yes	Infrequent	Infrequent

a Information obtained from National General Practice Profiles (available at https://fingertips.phe.org.uk/profile/general-practice/data). Scoring: low = 1–3, medium = 4–6, high = 7–10.

b Indicating percentage of patient population who belong to black or ethnic minority groups.

c Information reported by GP. BME = black and minority ethnic. CAMHS = Child and Adolescent Mental Health Services. n/a = not applicable. TV&SM = Thames Valley & South Midlands.

### Participants

Twenty GPs were recruited to the study, via an e-mail invitation from their local NIHR Clinical Research Network. Theoretical saturation was reached by participant 18, after which two more interviews were analysed to ensure that no further evidence was being captured to enhance the conceptual categories. Participants were informed that the purpose of the study was to explore GPs’ experiences of anxiety disorders in children aged <12 years of age. Purposive sampling was employed at this stage to ensure that the sample represented a diverse range of patient, practitioner, and service characteristics, including the ethnic and socioeconomic profile of the patients served by the practice, GP age, sex, professional status in practice, whether the GP considered themselves to be ‘research active’ or not, and practice size and location (Table 1).

**How this fits in**

GPs are often the first port of call for parents concerned about their child’s mental health. GPs can feel ill equipped, however, to manage anxiety disorders in children. Reasons for this include a lack of confidence and inadequate training. There is little research in this area. This study describes GPs’ perspectives and experiences of supporting children with anxiety disorders across three themes: decision making, issues of responsibility, and emotional responses.

### Data collection and analysis

Individual interviews were conducted, using an interpretive approach, to explore GPs’ experiences with recognition, management, and access to specialist services for childhood anxiety disorders and recommendations for possible improvements. Interviews took place over the telephone and were audiotaped (range 22–53 minutes). A topic guide was developed on the basis of existing literature, including a recent systematic review,^35^ through discussions with the research team, who had expertise in childhood anxiety disorders and qualitative methodology, and a pilot interview with a GP. The topic guide was applied flexibly and revisions were made throughout the data collection period to ensure that emerging concepts informed subsequent interviews. Detailed field notes were made during both interviews and coding, and used to aid analyses and interpretation.

Thematic analysis was drawn on, using an inductive approach to identify, analyse, and report patterns in the data,^36^ using a constant comparative method, both between and within transcripts. The process began by transcribing the interviews verbatim, facilitating data familiarisation through repeated listening. Meaningful units of texts were organised using line-by-line coding, through the prism of the research question. Units of text were then considered in the context of the wider transcript, and in relation to the participant’s overall stance in relation to the topic, facilitated by the field notes taken during and immediately after the interviews. Codes were organised into groups and named on the basis of analysis. These groups were revised and refined numerous times until they formed themes. As part of the coding process, codes and themes were regularly presented to the wider research team, who contributed to their refinement and revision. All data were coded and used until the final stage, in which some items deemed irrelevant to the research question were not included. NVivo 10 facilitated the analysis.

## RESULTS

**Figure 1.**
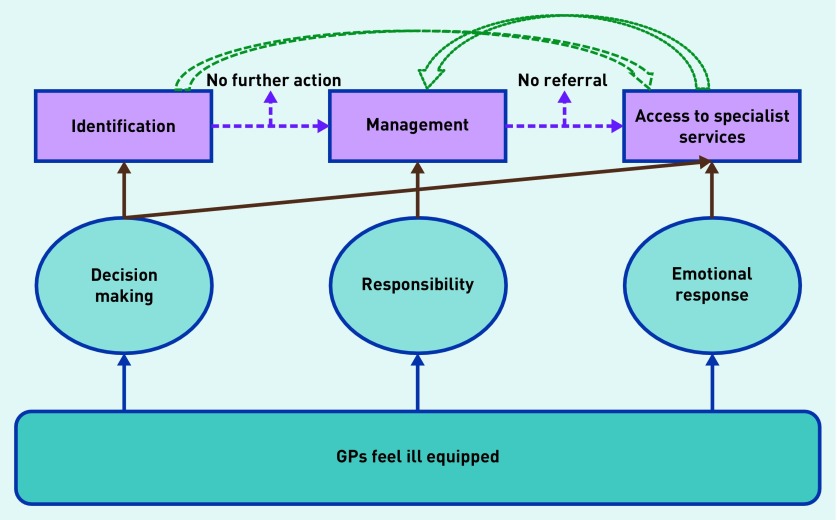
***Embedding emergent themes in the primary care process.***

Identified codes reflected three main themes: decision making, responsibility, and emotional response. Themes were initially identified regardless of where they occurred in the primary care process, then retrospectively organised to illustrate their impact on identification, management, and access to specialist services (Figure 1). The results are presented in terms of where the themes fitted within the primary care process.

The feeling of being ill equipped recurred across all three themes, having an impact on how GPs made decisions about childhood anxiety, in terms of identification and accessing specialist services. It also had an impact on the level of responsibility a GP felt in terms of managing this disorder and their emotional response to restricted access to specialist services.

### Identification

#### Decision making: uncertainty

GPs described uncertainty in identification, relating to a sense that anxiety disorders in children are particularly complex:

‘… not sure that it’s a disorder that can have very clear boundaries.’

(GP12)

This often induced GPs to seek further help from relevant professionals:

‘… would be able to have an inkling but to say a firm diagnosis, I would probably require assistance.’

(GP20)

This uncertainty seemed to be exacerbated by children often presenting with physical symptoms that might be accounted for by other conditions. Most GPs did not use any screening tools to identify a suspected anxiety disorder, and, although some expressed doubts, many identified a tool as something that could enhance their decision making, especially if it was:

‘… fairly straightforward … and well validated by research.’

(GP4)

In the absence of access to specialists (see ‘Access to specialist services’ further on), collaboration with colleagues increased some GPs’ confidence with recognition. Being a parent also aided identification in terms of understanding the range of typical child development and recognising abnormal behaviour.

#### Decision making: prioritisation

GPs discussed how anxiety disorders were often not prioritised as highly as other childhood conditions, and possibly not seen as disorders in their own right:

‘… it can sometimes be a challenge to pinpoint that down and focus on that individually.’

(GP20)

For GPs, physical illness generally took priority over mental illness, which often seemed to be driven by desires of the parents:

‘… they come in … and you know that this is anxiety related but … before labelling I would always make sure that there is no other physical reason going on.’

(GP19)

The prioritisation of physical over mental health may, in part, reflect the time restrictions placed on GPs, as identification was seen as something that takes some time:

‘… the problem is, we obviously see 20 children in the morning with coughs and colds, we don’t have the time.’

(GP8).

GPs differed in whether they were routinely looking out for anxiety disorders in children, with some saying:

‘… pretty aware of it and try to incorporate in my consultation if I have time.’

(GP8)

However, others were less aware:

‘… we’re not really trained to look out for it as much if I’m honest.’

(GP3)

This appeared to relate to their beliefs about the prevalence of childhood anxiety disorders, which ranged from uncommon to a growing problem.

Many GPs reported that they did not commonly encounter anxiety disorders and invoked this as a particular barrier:

*‘… because anxiety disorders in children particularly aren’t that common then it’s not something that we would necessarily know a lot* [about]*.’*

(GP4)

GPs also felt that there was a general lack of awareness about this disorder:

‘… compared with some of the more noticeable ones like autism or ADHD.’

(GP20)

But believed that increasing media and public awareness *‘… would be really good’*. (GP11)

### Management

#### Responsibility: role of GP

Some GPs did not see primary care as the appropriate place to manage childhood anxiety disorders:

‘I can’t tell them not to worry you know, they need to address the background problem, and if there isn’t a background problem and it’s a primary concern … then I do need some specialist input for that.’

(GP7)

These GPs saw their role mainly as a referrer. This attitude seemed to have been passed on from medical training:

*‘…* [the message was] *“oh well, as and when you see children with anxiety or depression, it’s specialist area” … so you know you’re not doing much except referring on anyway, [resulting] in a bit of a skills gap.’*

(GP3)

GPs also highlighted a lack of confidence managing childhood anxiety disorders, which was often linked to a lack of training in child and adolescent mental health:

‘I did a psychiatric rotation … I think we had one half day in child and adolescent mental health and that was the sum of it.’

(GP3)

Concern about getting it wrong was also highlighted:

‘… we don’t want to mess anything up’

(GP8)

There was a feeling among some that they would be uncomfortable weighing in on what is seen as a private family matter:

‘… start to feel like I’m going a little beyond my remit because … you start to feel a little bit like you’re giving parenting advice … that’s quite a personal thing.’

(GP7)

Increased training in child and adolescent mental health was seen as a potential route to improving GPs’ practice:

*‘would be really important* [if] *you had some sort of exposure* [during psychiatric training] *in knowing how to diagnose and initial management of anxiety disorders in kids.’*

(GP13),

This would also reduce referrals to CAMHS:

*‘… if all GPs had skills and confidence then that would take out referral* [for] *a lot of the kids who might not need it, which would mean that the more severe problems or the more difficult to treat problems … could get seen sooner or for longer.’*

(GP16)

In addition, increased support was seen as a facilitator:

‘… things that might be perfectly manageable within primary care … I think that those skills and the confidence to do it, would be improved by support.’

(GP15)

The extent to which GPs felt managing childhood anxiety was part of their role influenced their views about training and support. Some GPs did not feel that further training was required as they thought it was:

‘… possibly a bit of a waste of time … because when it comes to the real world as a GP you wouldn’t be initiating treatment anyway.’

(GP3)

Other GPs felt quite comfortable managing this condition:

*‘I do* [feel confident] *‘cause they’re common and a lot of them are about reassurance and common sense and just letting people talk and so that’s what GPs do.’*

(GP16).

Some GPs, particularly those with a special interest in this area, explained that they would like to have an increased role, and saw this as part of their GP identity:

‘I like to manage as many things as I can and I think most GPs do really because you get satisfaction from doing things yourself.’

(GP4)

Both GPs who did and did not want an increased role highlighted the benefit of having GPs who specialised in this area:

‘… who are comfortable in dealing with children and families and perhaps might have a few more answers or ideas.’

(GP10)

This type of involvement would require GPs to go beyond their normal 10-minute consultations, something that not all GPs are prepared to do:

‘… whether people are prepared to do that partly depends on the practice and what they’re prepared to do.’

(GP16)

Despite their best intentions, other pressures in primary care were sometimes cited as preventing GPs from having a role:

*‘…* [it] *will depend on what else is going on in primary care, and the other pressures and workload … so all the changes in other parts of the system might impact on what people do in childhood anxiety.’*

(GP16)

Time restrictions also prevented some GPs from prioritising management of childhood anxiety disorders:

‘… with such short appointments, I don’t think it would be appropriate for us.’

(GP?)

However, this view was not held by others if they believed that they could increase their role. GPs reported a general lack of awareness of any suitable management tools and would like tools to be available:

‘… if you could work through something with the patient and increase their awareness, I think I’d have increased confidence.’

(GP1)

The experience of being a parent was cited by numerous GPs as enhancing their ability to manage childhood anxiety disorders, as it allowed flexibility in their approach and helped them to empathise and relate to the parents that they were working with:

‘I don’t think I’d be as good at my job as a GP working in the field of mental health with children if I wasn’t a parent.’

(GP10)

#### Responsibility: responsibility of others

There was a concern among some GPs that nobody seems to be taking responsibility for this group of patients:

‘… it ends up on GP’s door.’

(GP19)

Many felt that other services and organisations could be doing more and a lack of integration was causing problems:

*‘… as a GP I feel very separate from all of that* [specialist services]*, whereas it shouldn’t be really.’*

(GP10)

Many GPs believed that school was the best place to manage anxiety-related conditions:

‘… ‘cause children spend most of their hours of their days there.’

(GP10)

And many thought that schools should receive more training to increase their capabilities:

*‘… training could* [help] *the school nurses in particular … so that* [anxiety] *can also* [be] *picked up at a school at a much earlier level, where the parents may not pick it up.’*

(GP17)

Although some GPs didn’t feel that close contact with schools would be possible, collaborations with schools, where they existed, seemed to be helpful.

### Access to specialist services

#### Emotional response: frustration with specialist services

Most GPs described a general frustration relating to difficulties accessing CAMHS, mostly because of slow responses and rejected referrals. Although frustration accessing specialist services was very commonly mentioned, however, there were divergent views, with one GP stating that they had never experienced rejected referrals. A sense of general confusion among the GPs regarding the structure of CAMHS exacerbated this problem:

*‘… it’s become so complicated that nearly always I get a letter talking* [to] *me about tier something or other, and I don’t understand what they’re talking about.’*

(GP16)

They stated that it changed regularly, which led to GPs feeling unsure of what CAMHS offered:

‘… they’re a team out there, I don’t know what they look like … I have no idea, it’s just a name, you know at the bottom of paper, we don’t really talk to them.’

(GP10)

Most GPs described CAMHS referral criteria as confusing:

*‘…* [CAMHS could] *feedback better and actually clarify what exactly they could see or not, because it doesn’t seem to be very clear to me.’*

(GP8)

This seemed to be particularly true of problems that were not straightforward:

‘… sometimes that can almost be a barrier because they wouldn’t fit within that box, they tend to say “no we won’t see them”.‘

(GP20)

A commonly expressed frustration was that children should not have to be at extreme levels of severity to access help:

‘… the horrible thing is waiting until they get bad enough and then you’ve got to send them back again once you’d have preferred to not have to let it get to that point.’

(GP13)

GPs generally felt that they did not make inappropriate referrals, and, thus, rejection was particularly frustrating:

‘… with children you think it would be more important that if a GP was worried they would take the referral, but they don’t.’

(GP18)

This was sometimes seen as *‘… a bit of a slap in the face’*. (GP13)

#### Decision making: utility of identification to access specialist services

Restricted access also sometimes prevented GPs from initially identifying childhood anxiety difficulties in the first place:

‘… it’s leading somebody down a path and then saying well actually this is what we have but there’s no set service for this and no set treatment.’

(GP17)

Other GPs felt that, despite the difficulties accessing services, they had no choice in the matter:

‘… otherwise you wouldn’t become a doctor, you just cannot ignore it, it’s just not possible, but your heart sinks.’

(GP19)

GPs’ beliefs about the efficacy of current treatments for anxiety also influenced this decision; some considered CAMHS to be effective, although others were less convinced. This lack of confidence sometimes reflected a general lack of understanding of what treatments might be available for child anxiety disorders:

‘… I’m not really quite sure what there is in terms of therapies or counselling, that is actually available, that is validated for children that age.’

(GP5)

And there was a call for more information to be available. Those who believed that outcomes for childhood anxiety disorders could be serious were particularly keen on accessing interventions at an early stage:

*‘… it’s awful, if we wait until they’re adults, they get counselling referral, but I think actually the glass is already broken so I think it’s* really *important to do it early on.’*

(GP8)

#### Emotional response: helplessness

GPs often described a feeling of helplessness:

‘… what sometimes happens is that these poor children and their families tend to get passed from pillar to post and then 6 months down the road they end up coming back to see you and they say “we’ve been signposted to all these different things, basically the last person we saw said to come back and see your GP”, so they’ve wasted all this time, they haven’t got any answers to their problems.’

(GP5)

This often had an impact on the GPs themselves:

‘… don’t really know what to do because we’ve referred because we’re out of our depth and then it gets rejected.’

(GP13)

This left GPs *‘stuck’* (GP10, GP19, GP20) and unsure of how to proceed in this instance, struggling to manage families’ expectations. Access to a specialist, prior to making a formal referral, was mentioned as something that could decrease this sense of helplessness:

*‘… sometimes me chatting to a* [specialist] *might be enough to make me confident about what to do and that’s quite hard to get actually but that for a lot of things that could make a real difference.’*

(GP16)

Although some GPs highlighted the practical restrictions associated with having close contact with CAMHS as:

‘… they’re only one of many people that we refer to.’

(GP9)

Increased communication and collaboration with CAMHS were often mentioned as potential facilitators:

‘… would increase confidence if it was a bit more joined up.’

(GP13)

#### Decision making: parental impact

Parents appeared to be particularly influential in GP decision making about accessing specialist help for childhood anxiety disorders, reflecting the young age of the children being discussed. GPs highlighted that parents differ in the degree to which they accept an anxiety disorder diagnosis:

*‘… they* [parents] *can sometimes be resistant to that type of diagnosis.’*

(GP18)

And this caused a barrier for some, requiring careful handling:

‘… there is a reluctance to actually engage the parents in with that conversation.’

(GP17)

GPs who worked in areas with a more ethnically diverse patient population saw this as a big issue:

‘… certainly with a Southeast Asian or Eastern European population, they’re more keen on getting a physical diagnosis … rather than trying to accept that this might be a manifestation of an underlying mental health disorder.’

(GP17)

Other GPs, however, once the diagnosis had been explained to parents, would generally be accepting as:

‘… parents only want what’s best for their child.’

(GP20)

GPs were also very influenced by the parent’s wishes. Indeed, they were often viewed as the most important factor:

‘… if people didn’t want to be referred, then I wouldn’t refer them.’

(GP11)

This could involve listening to the parents’ desires, above and beyond their professional opinion:

*‘… inevitably, and I think often, those that shout loudest get the most, which you know, we try to be fair and we try to even that out, but if I felt a child was, not necessarily needing secondary care but the family were overly concerned and were pushing for a referral, I would probably* [go] *along with that.’*

(GP1)

However, most GPs felt that parents generally had a valid reason for wanting a referral:

‘… because it’s not something that I perceive parents bringing their children to the GP about lightly, I would have a pretty low threshold for referring onwards.’

(GP12)

## DISCUSSION

### Summary

This study highlights that GPs feel ill equipped to manage and effectively support childhood anxiety disorders, across all stages of the primary care process. Issues around decision making, who should be taking responsibility, and a GP’s own emotional response appear to have an impact on identification, management, and referral. Particular issues that appear to hinder identification of childhood anxiety disorders include uncertainties around diagnosis and the prioritisation of physical illness. Time was also often mentioned as a barrier, which is likely to influence how GPs prioritise different conditions.^37^ In terms of management, GPs responded in very different ways, reflecting discrepancies and doubt regarding the GP’s role. Where specialist help is required, parental pressure and views about the likelihood of accessing support play a key role in determining whether a GP will pursue specialist services, a process that can often leave GPs feeling helpless and frustrated.

### Strengths and limitations

Purposive sampling led to inclusion of GPs from a range of different training backgrounds, locations, and patient populations, although the lack of sessional GPs is a shortcoming of the study. This was facilitated by use of telephone interviews, which provided flexibility for GPs to participate within their busy working lives; however, nuances of facial expression and body languages could not be detected. GPs varied widely in their experience of child anxiety disorders, presenting the potential limitation that they may have talked hypothetically at times or with reference to their knowledge of this disorder in older age groups. Finally, GPs may have wanted to present themselves in a particular light, or carried assumptions about the researcher, which may have shaped their responses in different ways.

### Comparison with existing literature

Consistent with the findings of Roberts *et al* 30^,^^38^ regarding GPs’ perceptions of managing mental health disorders in teenagers, uncertainty was a key theme in relation to GP decision making about identification of childhood anxiety disorders. Like Hinrichs *et al*,^39^ this study also found that GPs experienced high levels of frustration in relation to accessing services for youth mental health problems and echoed the need for increased access to assessment tools and ongoing training for GPs. The present study is novel, however, in its focus on childhood anxiety disorders, rather than mental health in general or externalising conditions in children and/or young people. Many of the findings from this study confirm the barriers that have been found in studies that look at childhood mental health more generally;^35^ however, aspects that may affect GPs with regards to anxiety more specifically include issues around the utility of identifying this disorder, and the impact that being a parent themselves has on the experience of managing childhood anxiety disorders in primary care. The focus of this study on a young age group brought out the key role that parents play in GP decision making around this condition.

### Implications for research and practice

This study provides in-depth insight into issues GPs face in managing childhood anxiety disorders. Given the variation in GPs’ views and experiences, future research should investigate the contexts in which specific barriers do and do not occur to identify when, where, and how to implement targeted solutions. For example, those in larger surgeries may have more experience with this condition, which could potentially increase the importance and priority they afford to it. Given the high prevalence of anxiety disorders, the fact that many GPs report that they do not encounter this condition highlights an important issue: namely, that many parents of children with anxiety disorders may not be seeking help via their GPs, or that their difficulties may not be being recognised, which may result from parental uncertainty regarding how and where to seek help.^40^ That GPs are so influenced by parental wishes when making decisions about this condition highlights the need to ensure that parents are competent at identifying when a problem may be occurring with their child, and clearly understand that their GP’s surgery is an appropriate place to bring their child.

The stand-out message from the data was that GPs feel ill equipped to deal with childhood anxiety disorders. In line with a number of influential policy documents,^41^^,^^42^ this study provides support for the need for medical training to include greater emphasis on children’s mental health, at least for a subgroup of specialist GPs (as recommended by, for example, the *Chief Medical Officer’s Annual Report*, 2012)^43^ who might have more time allocated to work with affected families. Furthermore, better integration between primary and specialist CAMHS may help to deal with the problem which is that GPs feel separate from, and unsupported by, specialist services, by promoting a better understanding of what services are offered and providing opportunities for GPs to seek advice (potentially enabling GPs to manage these conditions and reducing the need for specialist referrals). The proposed expansion of primary care-based services to include child mental health professionals within general practices would facilitate this and allow specialist services to concentrate on more severe and/or complex cases.

## Appendix 1. Interview topic guide

### Background

1. Do you feel you personally have a clear concept of what an anxiety disorder is?
2. Do you believe there are differences between adults and children in terms of anxiety disorders?
3. What do you think the long-term outcome would be for a child with an anxiety disorder?
  - What if they do not receive specialist help (for example, pass with time, carry on, lead to other things?)?

#### Recognition

1. Do you see many children with anxiety symptoms?
  **If they say that they usually see teenagers, ask, ‘Do these teenagers indicate how long they have been suffering?’*
  - About how many a year?
  - How do they typically present?
  - Are the parents and/or child generally suspecting an anxiety disorder when they visit the practice?
2. Do you routinely look out for anxiety disorders in children?
  - Are there particular signs and symptoms you look out for?
  - How confident do you feel about recognising the symptoms?
  - What would you do if you suspected an anxiety disorder but it wasn’t the presenting problem?
  - How do you code anxiety in your notes?
  - Are you aware of any tools available to help screen for and make decisions about anxiety disorders in children?
  - Do you use any of these tools?
    - If yes, why? If no, why not?
    - Do you ever use any online resources to help screen for anxiety disorders?

#### Management

1. How confident do you feel about managing anxiety disorders in children?
  - What influences how confident you feel?
  - Are you confident discussing the topic with the child?
  - Would anything increase your confidence?
  - What influences the management of your child’s anxiety disorder?
  - What do you draw on when you manage anxiety disorders?
  - Are you comfortable giving advice to parents in terms of managing anxiety disorders?
    - What sort of advice do you give?
      **Examples might be useful here*

#### Further s/teps

1. What is your decision-making process if you suspect an anxiety disorder?
  - Would you refer the child to a specialist service?
    - If yes, why? If no, why not?
    - If you don’t refer, how do you manage the child at that stage?
    - Use tools such as books, apps?
2. If you do choose to refer, when (for example, first/second visit?) does this usually occur?
  - What percentage of children do you refer onwards?
3. How severe would a child have to be for you to find it appropriate to refer the child onwards?
4. Are you influenced by any child and family factors?
  - *Prompts re. parents*: for example, pressure to refer, beliefs about parents’ ability to deal with issue
  - *Prompts*: do you think there are any particular family characteristics or circumstances that create a higher risk for anxiety disorders?
  - *Prompts re. children:* for example, severity, burden of normal life
    *Examples might be useful here
5. Do you ever prescribe medication for the child’s anxiety disorder?
  - When/why do you take this step?

#### Delivery of services in their area

I am interested in CAMHS generally but would also like to get a feel for how this service works for anxiety disorders

1. What specialist services are available locally for children with anxiety disorders?
  - Have you used them? How *many* times have you referred a child to CAMHS for an anxiety disorder?
    - Would you say more or less than five?
  - What is the process of referring onwards in your area?
    - *Prompts:* structure.
  - How do you find this process?
  - If not, should there be?
  - What is the first point of call?
  - What are the subsequent services?
2. Are you aware of the funding source for these services?
3. What is your opinion of specialist services for children with anxiety disorders?
  - How does it work in comparison with other services (for example, adult mental health services?)
  - What kind of feedback do you get from patients/family going through these services?
  - Do you think CAMHS are more or less likely to take anxiety disorders compared with other childhood mental health issues?
4. How would you describe your relationship with these services (*or CAMHS*)?
  - How do you feel about this relationship?
  - Is it important in managing childhood anxiety disorders?
5. Do your referrals generally get accepted?
  - What do you think about the feedback you get on referrals that are not accepted?
  - Do you continue to have contact with the family when the child is proceeding through CAMHS?

*Reinforce if situations are applicable to ANXIETY DISORDERS

** With recommendations: What prevents it from being like that?

**** If CAMHS has not come up at this point, mention it:*

1. *Do you ever refer a child with an anxiety disorder to CAMHS or PCAMHS?*
  - Follow with questions 2, 3, and 4 from above.

#### Recommendations

1. What do you think would aid the recognition of anxiety disorders in general practice?
  - Tools: What kind of tool would be useful for you to use for screening in primary care (for example, format, time-taken, no. of items)?
2. What do you think would aid the management and treatment of anxiety disorders in primary care?
  - Would you be interested in online tools for the management of anxiety disorders in primary care?
3. What do you think would improve the referral pathway between primary care and other specialist services? (*& specifically about CAMHS?*)
4. Are there any other things you think would help or anything that you feel we haven’t mentioned that would be useful to comment on?
